# Dynamics of macronutrient self-medication and illness-induced anorexia in virally infected insects

**DOI:** 10.1111/1365-2656.12127

**Published:** 2013-09-02

**Authors:** Sonia Povey, Sheena C Cotter, Stephen J Simpson, Kenneth Wilson, Sonia Altizer

**Affiliations:** 1 Lancaster Environment Centre, Lancaster UniversityLancaster, LA1 4YQ, UK; 2 School of Biological Sciences and the Charles Perkins Centre, University of SydneySydney, NSW, 2006, Australia

**Keywords:** diet, geometric framework, immunity, Lepidoptera, nucleopolyhedrovirus, parasite, pathogen, resistance, *Spodoptera exempta*

## Abstract

1. Some animals change their feeding behaviour when infected with parasites, seeking out substances that enhance their ability to overcome infection. This ‘self-medication’ is typically considered to involve the consumption of toxins, minerals or secondary compounds. However, recent studies have shown that macronutrients can influence the immune response and that pathogen-challenged individuals can self-medicate by choosing a diet rich in protein and low in carbohydrates. Infected individuals might also reduce food intake when infected (i.e. illness-induced anorexia).
2. Here, we examine macronutrient self-medication and illness-induced anorexia in caterpillars of the African armyworm (*Spodoptera exempta*) by asking how individuals change their feeding decisions over the time course of infection with a baculovirus. We measured self-medication behaviour across several full-sib families to evaluate the plasticity of diet choice and underlying genetic variation.
3. Larvae restricted to diets high in protein (P) and low in carbohydrate (C) were more likely to survive a virus challenge than those restricted to diets with a low P: C ratio. When allowed free choice, virus-challenged individuals chose a higher protein diet than controls.
4. Individuals challenged with either a lethal or sublethal dose of virus increased the P: C ratio of their chosen diets. This was mostly due to a sharp decline in carbohydrate intake, rather than an increased intake of protein, reducing overall food intake, consistent with an illness-induced anorexic response. Over time the P: C ratio of the diet decreased until it matched that of controls.
5. Our study provides the clearest evidence yet for dietary self-medication using macronutrients and shows that the temporal dynamics of feeding behaviour depends on the severity and stage of the infection. The strikingly similar behaviour shown by different families suggests that self-medication is phenotypically plastic and not a consequence of genetically based differences in diet choice between families.

## Introduction

By definition, parasites reduce the fitness of their hosts by diverting hosts’ nutritional resources for their own growth and reproduction and by causing other fatal or debilitating effects (Schmid Hempel 2011). To counter this threat and to minimize the costs of parasitic infection, multicellular organisms have evolved an effective immune system to recognize and attack invading parasites. But immune defences are costly; they can cause self-harm when triggered (Sadd & Siva-Jothy 2006) and also demand nutritional resources that could otherwise be channelled into growth and reproduction (e.g. Moret & Schmid-Hempel 2000; Siva-Jothy & Thompson 2002; Cotter, Kruuk & Wilson 2004).

The nutritional state of the host can affect its ability to fight and resist an infection (Chandra 1996; Lochmiller & Deerenberg 2000) such that increasing an organism’s access to resources can increase its resistance to parasites. For example, food-supplemented snowshoe hares (*Lepus americanus*) experienced reduced nematode prevalence compared to controls (Murray, Keith & Cary 1998), whilst experimental food restriction suppressed cell-mediated immunity in yellow-legged gulls (*Larus cachinnans*, Alonso-Alvarez & Tella 2001). Similarly, invertebrate studies have focused on the effect of nutrient deprivation or starvation on immune function and/or parasite resistance, with the consensus being that reduced resources compromise immunity (e.g. Moret & Schmid-Hempel 2000; Siva-Jothy & Thompson 2002; Ayres & Schneider 2009 but see Triggs & Knell 2012).

Often, energy is assumed to be the limiting resource that individuals must partition between traits, and indeed, mounting an immune response has been shown to increase the metabolic rate of both vertebrates (Demas *et al*. 1997) and invertebrates (Freitak *et al*. 2003). Despite the requirement for resources during an immune response, many animals display illness-induced anorexia, in which food intake is reduced immediately after an immune challenge (Kyriazakis, Tolkamp & Hutchings 1998; Adamo, Fidler & Forestell 2007). This may seem counter-intuitive but has been hypothesized to serve a number of possible functions, from reducing the risk of ingesting more parasites, to starving resident parasites of key macro- and micronutrients (see references in Kyriazakis, Tolkamp & Hutchings 1998; Adamo, Fidler & Forestell 2007). However, beyond the intake of energy, feeding comprises the ingestion of nutrients in particular ratios, which are allocated to different functions within the body, and there is good evidence that overingestion as well as underingestion of certain nutrients can be costly (Simpson *et al*. 2004; Raubenheimer, Lee & Simpson 2005; Cotter *et al*. 2011). Animals that would benefit from reducing the intake of a particular nutrient that favours parasite growth might be forced to decrease food consumption overall.

In lepidopteran larvae, resistance to parasites has been shown to depend on the relative amounts of macronutrients (protein and carbohydrate) in the diet and the diet that optimizes growth rates in uninfected individuals differs from the diet that optimizes the immune response (Lee *et al*. 2006; Povey *et al*. 2009; Cotter *et al*. 2011); thus, we might expect organisms to modify their intake based on their current nutritional requirements. This behaviour is known as self-medication, which Singer, Mace & Bernays (2009) define as ‘a specific therapeutic and adaptive change in behaviour in response to disease or parasitism’. It is generally recognized that verification of therapeutic self-medication must satisfy three criteria: (i) the behaviour should increase the fitness of infected individuals; (ii) it should decrease or have no effect on the fitness of uninfected individuals; and (iii) the behaviour should be specifically triggered by infection. There is evidence for therapeutic self-medication from several studies of vertebrates, most famously from chimpanzees that use plant-derived substances when infected with protozoan or helminth parasites (Huffman & Seifu 1989; Fowler, Koutsioni & Sommer 2007), and some experimental studies of livestock infected with gut nematodes using nitrogen-rich clover (Hutchings *et al*. 2003). There is also evidence from insect species for medicinal use of plant secondary compounds, such nicotine, pyrrolizidine alkaloids and iridoid glycosides (e.g. Krischik, Barbosa & Reichelderfer 1988; Christe *et al*. 2003; Castella *et al*. 2008; Singer, Mace & Bernays 2009). More recent studies have provided support for macronutrient self-medication in bacteria- or virus-challenged caterpillars (Lee *et al*. 2006; Povey *et al*. 2009). Although macronutrients are a ubiquitous part of the diet and their use is not restricted to self-medication, nearly all documented cases of self-medication involve increasing the amount of a nutrient or chemical that comprises some fraction of the normal diet (see Raubenheimer & Simpson 2009).

Implicit in the notions of self-medication and illness-induced anorexia is that changes in feeding behaviour should be dynamic, with the magnitude of the response depending on the stage of infection and the host’s capacity to resist or tolerate infection. To capture this dynamic, studies must control for differences in feeding behaviour prior to and during infection, that is, dietary preferences should be compared longitudinally *within* groups pre- and post-challenge. In addition, studies must consider the possibility that the capacity to self-medicate could have a significant genetic component, such that the magnitude, direction or timing of behavioural changes differs between families or genotypes (Lefevre *et al*. 2010).

Here, we assess the effects of dietary protein and carbohydrate balance on the outcome of infection with nucleopolyhedrovirus (NPV) in larvae of the African armyworm, *Spodoptera exempta*, and on the associated immune response. This is a natural host–pathogen interaction in sub-Saharan Africa (Graham *et al*. 2012), and *S. exempta* larvae feed on a wide range of graminaceous crops and pasture grasses that vary in their nutritional composition (Yarro 1984; Rose, Dewhurst & Page 2000). Using artificial diets to control macronutrient composition precisely, we measured the diet choice of individuals from different full-sibling families both before and after challenge with NPV, thus providing the strongest test yet for dynamical self-medication using dietary macronutrients. In so doing, we also examined the absolute amount of each macronutrient consumed to test whether sickness-induced anorexia, and/or selective intake of specific nutrients, occurred in response to infection. Our study tested the following specific predictions: (i) resistance to NPV will decline as the relative protein content of the diet is reduced, (ii) diet-related resistance to NPV will be associated with diet-related changes in immune function, providing a potential mechanism for changes in resistance, (iii) virus-challenged insects will prefer a diet rich in the macronutrient that favours NPV resistance in the short term and revert to diets similar to non-challenged individuals when the infection is under control, (iv) infection with NPV will trigger a short-term anorexic response, limiting the potential for further exposure to the virus or starving it of resources, and finally, (v) the degree of plasticity in the self-medication response will vary among full-sibling families, consistent with genetic variation in the trait.

## Materials and methods

### Insects and virus

*Spodoptera exempta* is a major crop pest throughout sub-Saharan Africa and feeds mostly on graminaceous plants, including the staple cereal crops maize, sorghum, millet, and rice, as well as on a diverse range of pasture grasses (see Rose, Dewhurst & Page 2000 for a full species list). As an outbreak pest species that frequently occurs at larval densities in excess of 100 per m^2^ (Rose, Dewhurst & Page 2000; Graham *et al*. 2012), *S. exempta* larvae will typically switch between plant species when feeding in mixed pastures, impacting on its growth and fitness (Yarro 1984). A continuous culture of *S. exempta,* originally collected in Tanzania, had been maintained at Lancaster University for 4 years (*c*. 48 generations) prior to the start of the experiments. More than 150 breeding pairs were established each generation to ensure high genetic variability. From the third-instar onwards, larvae were reared in isolation in 25-mL plastic pots containing a wheatgerm-based semi-artificial diet comprising *c*. 33% protein and 29% carbohydrate. Larvae were kept at a constant temperature of 25 °C under a 12 h: 12 h light: dark regime. All experiments were performed using newly moulted final-instar larvae.

The baculovirus *Spodoptera exempta* nucleopolyhedrovirus (SpexNPV) occurs naturally in *S. exempta* larvae and a recent study found that the prevalence of overt virus disease at high-density larval outbreaks in Tanzania ranged between 0% and 17% (Graham *et al*. 2012), though prevalences in excess of 90% have been reported in late-season outbreaks elsewhere (Rose, Dewhurst & Page 2000). Larvae become infected when they ingest vegetation contaminated by virus occlusion bodies (OBs) released from cadavers, though vertical transmission of virus is also common (Vilaplana *et al*. 2008, 2010). To generate sufficient virus for the experiments, virally infected cadavers were homogenized before being filtered through muslin and centrifuged at 3500 g for 5 min to remove larval debris. The supernatant was then pelleted by spinning for 20 min at 3000 g. The resulting pellet was resuspended in water and purified on a 50–60% discontinuous sucrose gradient at 30 000 g for 60 min. This purified virus was washed and pelleted three times in distilled water and spun at 10 000 g for 30 min. The purified virus was stored at −20 °C until needed. Dilutions needed for experiments were estimated using a Neubauer haemocytometer.

### Viral inoculations

Larvae were placed individually in Petri dishes (9 cm diameter), where they received a diet plug, of *c*. 100 mg, inoculated with 1 μL of either water (control), or a solution of SpexNPV (Grzywacz *et al*. 2008). The amount of virus administered was either an LD_50_ dose of 2000 OBs per aliquot or an LD_10_ dose of 400 OBs per aliquot (Povey 2008). The LD_50_ dose was used to quantify the effects of diet on virus-induced mortality, whilst the LD_10_ dose was chosen to elicit a strong and specific defence response whilst causing minimal mortality (Povey 2008). The diet plug used for the challenge contained 14% protein and 28% carbohydrate, which has been found to be the optimal diet for non-infected *S. exempta* larvae (Lee, Simpson & Raubenheimer 2004). The Petri dishes were placed on trays in plastic bags to prevent the diet plugs from drying out and only larvae that had consumed the entire plug were used in the experiments. After inoculation, larvae were transferred to one of the experimental diets described below.

### Artificial diets

The experimental diets (based on Simpson & Abisgold 1985) varied in their soluble protein and digestible carbohydrate content and have been used previously in studies using *S. exempta* (Lee, Simpson & Raubenheimer 2004). The protein portion of the diet consisted of a 3: 1: 1 ratio of casein, peptone and albumen, and the carbohydrate content consisted of a 1: 1 ratio of sucrose and dextrin. Other constituents of the diets were Wesson’s salts (2·4%), cholesterol (0·5%), linoleic acid (0·5%), ascorbic acid (0·3%) and a vitamin mixture (0·2%). The remaining portion of the diets was made up of cellulose, a non-nutritive bulking agent. The dry ingredients were suspended at a 1–6 ratio w/v in 1% agar solution. Five diets were used in total, in each case the protein and carbohydrate portion made up 42% of the final diet: 7% carbohydrate with 35% protein (7: 35), 14: 28, 21: 21, 28: 7, and 35: 7; the remaining 58% of the dry ingredient was indigestible cellulose.

#### Experiment 1: The effects of P: C ratio on larval survival and diet choice in insects challenged with a high dose (LD_50_) of NPV

The aim of this experiment was to ask how dietary protein-to-carbohydrate (P: C) ratio affects larval survival and to determine whether, when given a choice, virally challenged larvae actively select a diet that improves their survival.

#### No-choice treatment

Sixty larvae per diet treatment were used in this experiment, 20 control larvae and 40 challenged with an LD_50_ dose of NPV (2000 OB per larva). All larvae were inoculated upon reaching the final instar and randomly placed on one of five diets varying in P: C ratio from extremely carbohydrate-biased to extremely protein-biased: 7: 35, 14: 28, 21: 21, 28: 14 or 35: 7. Given a choice, healthy *S. exempta* choose a carbohydrate-biased diet (19: 23; Lee, Simpson & Raubenheimer 2004). Ten caterpillars (three control and seven virally challenged) were discarded as they failed to consume the inoculated diet plug. Fresh diet blocks were provided each day post-infection until the larvae had ceased feeding at the pre-moult stage. All deaths were recorded to the nearest day and checked for the presence of OBs, though viral loads were not quantified due to logistical constraints.

#### Self-selecting treatment

Sixty final-instar larvae were weighed to the nearest 0·001 g before being inoculated with either an LD_50_ dose of NPV (*n* = 32) or with distilled water (*n* = 28). After inoculation, larvae were placed in Petri dishes and given a choice between the two most extreme diets (35: 7 vs. 7: 35), to maximize the chances of detecting an effect of viral inoculation on diet choice. Diet blocks, each weighing between 0·7 and 1·3 g, were replaced daily until the larvae had ceased feeding at the pre-pupal stage. Uneaten food was dried to a constant mass in a desiccating oven. Consumption was calculated as the difference between the initial and final dry weight of each diet block. The initial dry weight of the blocks was estimated using regression of control blocks for each diet type (Lee *et al*. 2006). From the dry mass of food eaten, the amount of protein and carbohydrate consumed on each day was estimated. Deaths were monitored daily until all larvae had died or pupated; viral infection was confirmed by the presence of OBs.

### Experiment 2: The effects of P: C ratio on immune function and diet choice in insects challenged with a low dose (LD_10_) of NPV

This experiment tested whether immune responses were up-regulated in virally challenged larvae and how diets with different P: C ratios affected those responses. We used a low-dose viral challenge (LD_10_) to stimulate a strong defence response whilst minimizing mortality. We also performed a second choice test using this low viral dose to determine whether this was sufficient to change larval feeding behaviour. In addition, larvae from three full-sibling families were split across the treatment groups to test for genetic effects on diet choice and immune parameters.

#### No-choice treatment

On reaching the final instar, 160 larvae, 32 per diet treatment, were inoculated with either an LD_10_ dose of virus (400 OB per larva) or distilled water, as described above. Larvae were then provided with a diet block of one of the five chemically defined diets, as before. After being allowed to feed on the diets for 24 h, haemolymph was collected from the larvae. One larva died before haemolymph was collected and so was discarded from the experiment. Phenoloxidase (PO) activity, antimicrobial activity and haemocyte density were then measured for each sample (see below).

#### Self-selecting treatment

The methods for the self-selection treatment were as described in Experiment 1, with the following modifications: larvae were placed on their assigned diets for 24 h before viral inoculation. Larvae were given an LD_10_ viral dose and were provided with the choice between a 14: 28 diet and a 28: 14 diet. These ratios were chosen as we wanted to determine whether diet choice would be apparent even when the diets varied relatively little in their nutritional composition.

#### Antimicrobial activity

Antimicrobial growth inhibition assays were carried out as described in Povey *et al*. (2009) using an agar overlay technique (Rahalison *et al*. 1991) and the gram-positive bacterium *Micrococcus luteus*. Briefly, 1-μL samples of fresh haemolymph were pipetted directly into labelled holes on the agar plates, which were incubated for 24 h at 37 °C. Antimicrobial activity was measured as the radius of the clear zone of bacterial growth inhibition around the holes in the plate. Measurements were made using image pro plus software 4.1 (Media Cybernetics, Silver Spring, MD, USA).

#### Phenoloxidase activity and haemolymph protein levels

Phenoloxidase is a key enzyme in the prophenoloxidase cascade that generates highly cytotoxic quinones that can inactivate viral pathogens. The end-point of this melanization reaction is the production of melanin, which can kill macroparasites and viral-infected cells. Following haemolymph collection, samples for assaying PO activity were frozen at −80 °C until needed. PO activity and the amount of protein per sample were measured as described by Povey *et al*. (2009). Briefly, 6 μL of each haemolymph sample was mixed with 300 μL of phosphate-buffered saline; 100 μL of the resulting solution was pipetted in duplicate into a microtitre plate with 4 mm dopamine and absorbance measured at 492 nm over 10 min at 25 °C on a VERSAmax microplate reader (Molecular Devices, Sunnyvale, CA, USA). Haemolymph protein levels were determined using a standard curve created using a BSA standard (Bio-Rad, Hercules, CA, USA); 10 μL of the haemolymph sample was added to wells in a microtitre plate containing 200 μL of the dye reagent and the resulting colour measured at 600 nm.

#### Haemocyte density

Haemocytes are the immune cells of insects and are important effectors against parasites and pathogens, including baculoviruses (Strand 2008). Immediately after collection, 10 μL of each haemolymph sample was added to 10 μL of a 50: 50 ethylenediaminetetraacetic acid/glycerol solution (Cotter, Kruuk & Wilson 2004) and stored at −80 °C until needed. Haemocyte counts were performed by pipetting 8 μL of the haemolymph sample onto each side of an improved Neubauer haemocytometer (Hawksley, Sussex, UK, www.hawksley.co.uk). Haemocytes were counted in five non-adjacent squares on each side of the haemocytometer; these were then summed to give an estimate of the haemocyte density for each larva.

## Statistical analyses

### Experiment 1

Survival analyses were performed using accelerated failure time (AFT) models using the s-plus 6.2 (Insightful Corp., Washington, DC, USA) statistical package. These describe the relationship between the hazard function, or the risk of death, and a set of explanatory terms (Cox 1972). The hazard function is the instantaneous probability of death for an individual still alive. The interactive effects of *treatment* (virally inoculated or control) and *diet* (the percentage protein content of the diet) on the instantaneous death rates were considered. The choice data were analysed using restricted estimate maximum likelihood (REML) mixed-effects models in genstat 14 (VSN International, Hemel Hempstead, UK), with caterpillar ID included as a random effect to account for multiple measures on each individual.

### Experiment 2

Antimicrobial activity, PO activity and haemolymph protein levels were analysed using GLM in R (v2.13.1). PO activity, haemolymph protein levels and haemocyte density were log-transformed to obtain normally distributed data to meet the assumptions of the GLM. *Family* and *treatment* were included as factors and *diet*, as both linear and quadratic terms, were included as independent variables in the model. As for Experiment 1, the self-selecting data were analysed using REML mixed-effects models in genstat 14 in which caterpillar ID was included as a random effect to account for multiple measures on each individual. The three individuals that died from viral infection were excluded from the consumption data.

## Results

### Experiment 1: The effects of P: C ratio on larval survival and diet choice in insects challenged with a high dose (LD_50_) of NPV

#### No-choice treatment

**Figure 1:**
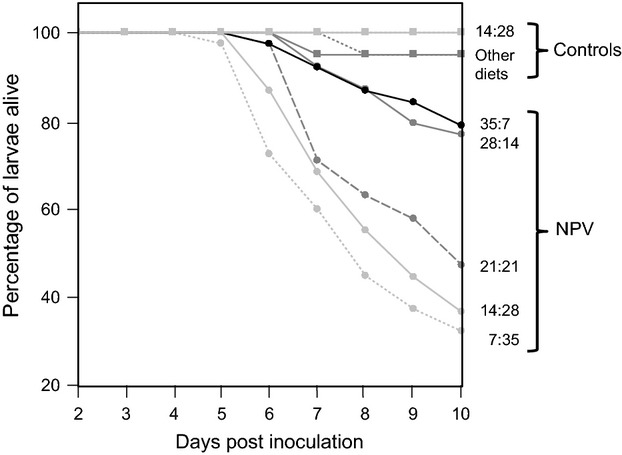
Survival curves for larvae restricted to one of five diets varying in their protein-to-carbohydrate ratios (P: C) and inoculated with either an LD_50_ dose of nucleopolyhedrovirus (NPV) or with water (controls). Data are taken from Experiment 1, no-choice treatment.

Larvae started to die from virus 4 days post-inoculation, and all larvae had either died or pupated by 10 days. Larval risk of death was affected by both viral inoculation (AFT model, *treatment*: 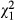 = 82·30, *P* < 0·0001) and the relative protein content of the diet (*diet*, 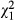 = 33·35, *P* < 0·0001). No other interactions were statistically significant. As expected, larvae inoculated with NPV had substantially lower survival than those in the control group (mean survival: control = 98%, NPV-challenged = 54%; estimate ± SE = −0·40 ± 0·09; Fig. 1). Whereas survival in the non-challenged insects was uniformly high (>95%) across diet treatments, in the virus-challenged larvae, survival increased with the ratio of protein to carbohydrates (estimate ± SE = 0·60 ± 0·01; Fig. 1), such that on the most protein-rich diet (35: 7), 79% of the virally challenged larvae survived, compared to just 33% on the most protein-poor diet (7: 35).

#### Self-selecting treatment

**Figure 2:**
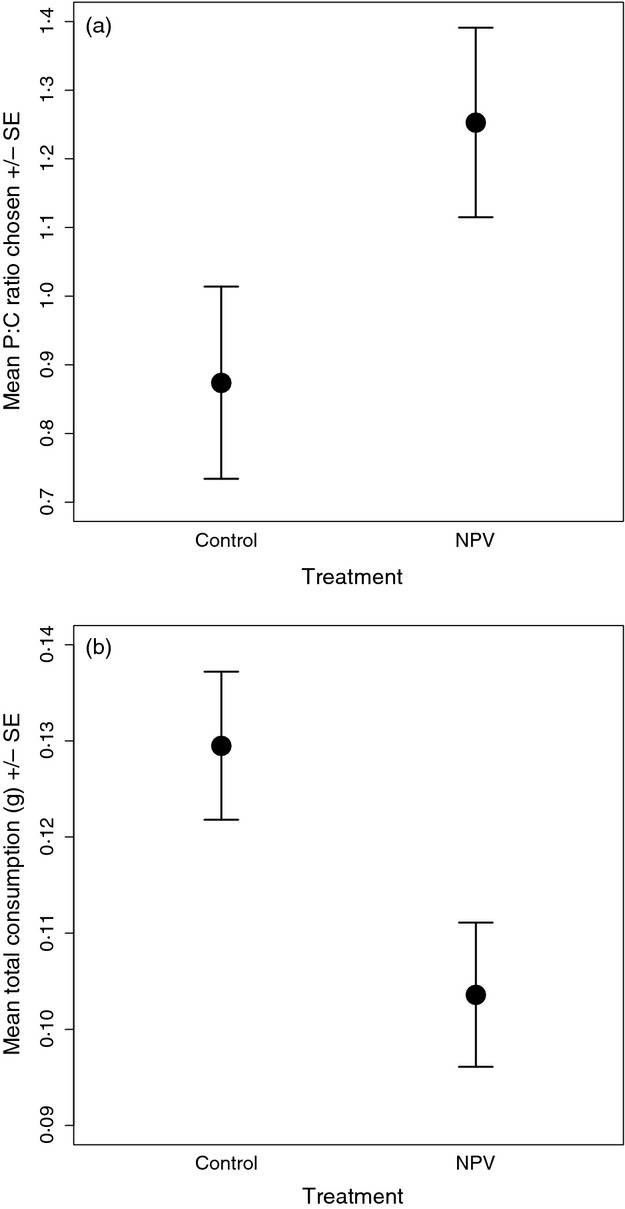
Effects of virus treatment on (a) the mean P: C ratio of the diet selected and (b) the total amount of food consumed. Larvae were inoculated with either an LD_50_ dose of nucleopolyhedrovirus (NPV) or with water (controls). Data are taken from Experiment 1, self-selecting treatment.

**Figure 3:**
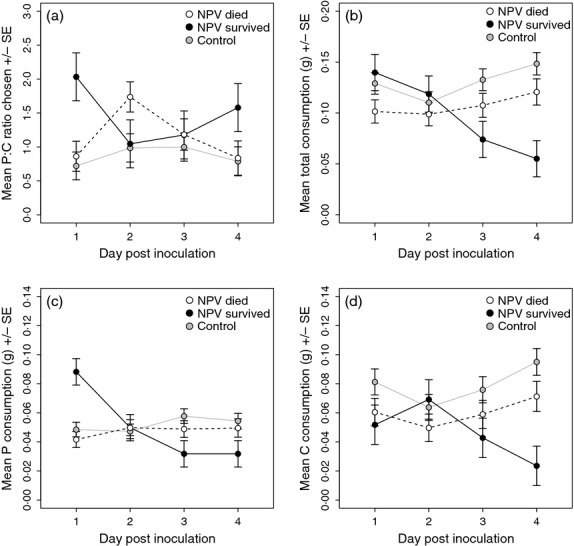
Effects of the outcome of infection (those that survived or died vs. controls) on (a) P: C ratio of the diet chosen, (b) the total amount of food consumed (c) the amount of protein consumed and (d) the amount of carbohydrate consumed. Data are taken from Experiment 1, self-selecting treatment.

Larvae that were inoculated with an LD_50_ dose of NPV chose a higher P: C ratio diet than larvae that were given water only (REML: *treatment*: *F*_1,55_ = 6·93, *P* = 0·011, Fig. 2a); there was no effect of time post-inoculation on diet choice and no significant interaction between these two factors (*day*: *F*_3,166_ = 1·44, *P* = 0·232; day*treatment: *F*_3,163_ = 0·54, *P* = 0·657). There was also no effect of larval weight on the P: C ratio of the chosen diet (*larval weight*: *F*_1,69_ = 2·19, *P* = 0·143). When we examined larvae that died from NPV separately from those that survived (giving three treatment groups: – control, NPV-survived and NPV-died), there was a significant interaction between day and treatment (day*treatment: *F*_6,159_ = 2·44, *P* = 0·028). Larvae that survived viral challenge showed an early shift towards a high P: C ratio diet on day 1 compared to controls, whilst those that later died from viral infection did not increase their P: C preference until day 2 (Fig. 3a).

Analysis of total food consumption, a measure associated with illness-induced anorexia, showed that larger larvae consumed more food than smaller larvae (*larval weight: F*_1,69_ = 10·26, *P* < 0·001). However, virally challenged larvae also ate significantly less than the controls (*treatment*: *F*_1,57_ = 11·33, *P* < 0·001; Fig 2b). As before, there was no effect of time post-inoculation on the daily amount of food consumed or a significant interaction between the two (*day*: *F*_3,167_ = 1·30, *P* = 0·278; day*treatment: *F*_3,163_ = 1·89, *P* = 0·134). Considering larvae that died from NPV separately from those that survived, there was a strong interaction between day and infection treatment (day*treatment: F_6,160_ = 4·31, *P* < 0·001; Fig 3b). Whilst control larvae and those that died from viral infection maintained a similar level of food consumption over the 4 days, those that survived viral challenge *decreased* their consumption as time went on (Fig. 3b).

These effects on the proportion and total amounts of the two foods eaten translated into differences in amounts of protein and carbohydrate eaten. Consumption of both macronutrients increased with larval weight (*larval weight*: P−*F*_1,68_ = 9·35, *P* = 0·003; C−*F*_1,69_ = 7·14, *P* = 0·009), but there were also significant interactions between infection treatment and time (day*treatment: P−*F*_6,158_ = 5·54, *P* < 0·001; C*−F*_6,160_ = 2·92, *P* = 0·010; Fig. 3c, d). Controls and those that died of infection maintained their protein intake during the 4 days post-inoculation. In contrast, survivors ate much higher levels of protein on day 1, then decreased consumption steadily over the next 3 days (Fig. 3c). Carbohydrate consumption, in contrast, was slightly higher in the controls on day 1, but whereas consumption tended to increase over time for controls and those that died of infection, it fell off significantly in those that survived infection (Fig. 3d).

### Experiment 2: The effect of P: C ratio on immune function and diet choice in insects challenged with a low dose (LD_10_) of NPV

#### No-choice treatment

**Figure 4:**
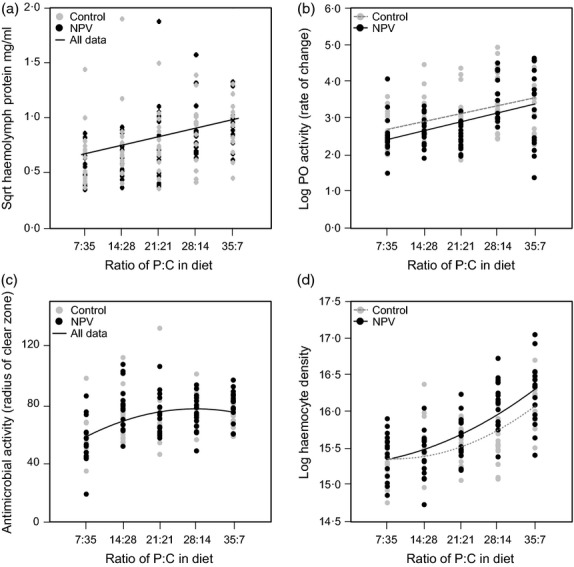
Effects of virus treatment and larval diet on (a) haemolymph protein levels, (b) haemolymph phenoloxidase activity, (c) haemolymph antimicrobial activity and (d) haemocyte density. Larvae were restricted to one of five diets varying in their P: C ratio following inoculation with either an LD_10_ dose of nucleopolyhedrovirus (NPV) or with water (controls). Data are taken from Experiment 2, no-choice treatment.

Mortality in this experiment was 8%, and the analysis excludes larvae that subsequently died of virus infection. Haemolymph protein levels increased with the amount of protein in the diet, such that highest levels were at P: C = 35: 7 (GLM: *diet*: *F*_1,157_ = 25·13, *P* < 0·0001; *diet*^2^: *F*_1,155_ = 0·15, *P* = 0·70; Fig. 4a). However, protein levels did not respond to NPV challenge or the interaction between viral treatment and dietary protein intake (*treatment*: *F*_1,156_ = 1·17, *P* = 0·28; treatment*diet: *F*_1,151_ = 1·51, *P* = 0·22; treatment*diet^2^: *F*_1,150_ = 0·12, *P* = 0·72; Fig. 4a). There was no significant variation between families in haemolymph protein levels (*family*: *F*_3,152_ = 0·50, *P* = 0·68), and none of the interactions with family were significant.

Phenoloxidase activity also increased with the protein content of the diet and peaked at P: C = 35: 7 (GLM: *diet*: *F*_1,157_ = 31·60, *P* < 0·0001; *diet*^2^: *F*_1,152_ = 0·20, *P* = 0·65; Fig. 4b), with virus-treated insects exhibiting a small, but significant, reduction in PO activity (*treatment*: *F*_1,156_ = 4·69, *P* = 0·032; Fig. 4b). The interaction terms were not significant (treatment*diet: *F*_1,151_ = 0·11, *P* = 0·74; treatment*diet^2^: *F*_1,150_ = 0·64, *P* = 0·42), and there were no family effects (*family*: *F*_3,153_ = 1·02, *P* = 0·38) nor any significant interactions between *family* and other terms in the model.

Antimicrobial activity increased nonlinearly with the protein content of the diet (GLM: *diet*: *F*_1,153_ = 25·72, *P* < 0·0001; *diet*^2^: *F*_1,153_ = 9·69, *P* = 0·002; Fig. 4c), peaking on a diet that was marginally protein-biased (P: C = 28: 14). However, antibacterial activity did not depend on NPV challenge (*treatment*: *F*_1,149_ = 0·32, *P* = 0·57; treatment*diet: *F*_1,148_ = 0·57, *P* = 0·45; treatment*diet ^2^: *F*_1,147_ = 0·007, *P* = 0·93), family group (*family*: *F*_3,150_ = 1·86, *P* = 0·14), or interactions with *family*.

Haemocyte density increased nonlinearly with the protein content of the diet, but peaked at P: C = 35: 7 (GLM: *diet: F*_1,153_ = 111·06, *P* < 0·001; *diet*^2^: *F*_1,153_ = 4·27, *P* < 0·001; Fig. 4d). However, in this case, being challenged with a low dose of NPV 24 h previously resulted in a stronger increase in the density of haemocytes in the haemolymph with increasing protein content of the diet (treatment*diet: *F*_1,153_ = 4·27, *P* = 0·04; treatment*diet^2^: *F*_1,152_ = 0·36, *P* = 0·55). There were no significant differences between families (*family*: *F*_3,151_ = 1·14, *P* = 0·33), and none of the interactions with *family* were statistically significant.

#### Self-selecting treatment

**Figure 5:**
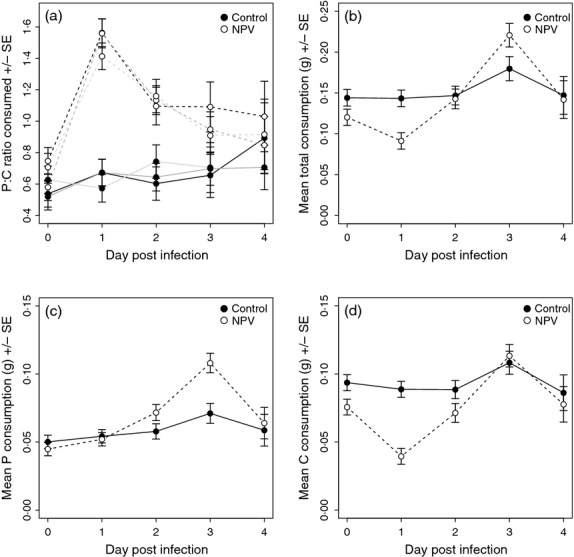
Effects of virus treatment on (a) the mean P: C ratio of the diet selected, (b) the total amount of food consumed, (c) the amount of protein consumed and (d) the amount of carbohydrate consumed by virus-challenged and control larvae on each day of the experiment. For figure (a), separate lines are plotted for each family to illustrate the similarity in diet choice across genotypes. Data are taken from Experiment 2, self-selecting treatment.

Before inoculation, both virus-challenged and control larvae chose a P: C ratio that was significantly carbohydrate-biased (Fig. 5a). However, following the challenge, the two treatment groups differed markedly in how their P: C diet choice changed over time (REML: day*treatment: *F*_4,210_ = 22·35, *P* < 0·001). The P: C ratio chosen by control larvae on the day following inoculation was carbohydrate-biased (mean P: C ratio = 1: 1·5) and increased moderately over time, whereas virus-challenged larvae increased their P: C ratio immediately after virus challenge to a strongly protein-biased diet (mean P: C ratio = 1·5: 1). This ratio then fell gradually over the next 3 days until the final ratio chosen was not significantly different from that of control larvae. Diet was not affected by *larval weight*, *family*, or any of their interactions (*F* < 0·55, *P* > 0·46).

Total food consumption also varied significantly between control and virus-challenged larvae. Before being inoculated, both virus-challenged and control groups consumed a similar amount of food (Fig. 5b). Food consumption differed significantly among families (REML: *family*: *F*_2,73_ = 4·35, *P* = 0·009), and heavier larvae ate more food (*larval weight*: *F*_1,69_ = 7·44, *P* = 0·008). Following the virus challenge the two treatment groups differed in total food consumption over time (day*treatment: *F*_4,191_ = 4·35, *P* = 0·002). Whilst control larvae ate a similar amount of food each day (Fig. 5b), the virus-challenged larvae *decreased* their food consumption immediately following challenge and then *increased* it steadily. By day 4, food consumption was the same for both groups (Fig. 5b). None of the other interaction terms were statistically significant (*F* < 1·73, *P* > 0·094).

Consumption of the two macronutrients also exhibited temporal variation and a significant effect of treatment, with the temporal change in nutrient consumption differing between control and NPV-challenged caterpillars (P−day*treatment: *F*_4,194_ = 3·23, *P* = 0·014; C−day*treatment: *F*_4,189_ = 6·15, *P* < 0·001; Fig. 5c, d). Whilst protein consumption gradually *increased* in virus-challenged insects relative to controls on days 3 and 4 post-inoculation, carbohydrate consumption *decreased* significantly on day 1 before returning to pre-inoculation levels thereafter. Consumption increased with larval weight (REML: *larval weight*: P−*F*_1,69_ = 5·90, *P* = 0·018; C−*F*_1,68_ = 7·69, *P* = 0·007) and differed among families (*family*: P−*F*_2,73_ =4·25, *P* = 0·018; C−*F*_2,72_ = 4·86, *P* = 0·010).

## Discussion

Here, we provide the clearest evidence to date for therapeutic self-medication, *sensu* Singer, Mace & Bernays (2009), using dietary macronutrients. Consistent with this phenomenon, *S. exempta* larvae challenged with a high (LD_50_) dose of NPV chose a diet that was rich in protein (containing *c*. 50% more P than C) compared to that of uninfected control larvae, which chose a diet that was carbohydrate-biased (*c*. 50% more C than P). By choosing a relatively protein-rich diet, NPV-challenged insects improved their survival prospects from <40% on foods containing the most carbohydrates (P: C = 7: 35 and 14: 28) to around 80% on the most protein-rich foods (P: C = 28: 14 and 35: 7). In this and previous studies, the survival of non-infected larvae was high and independent of P: C ratio, but larval growth rate and overall performance (survival × larval growth rate) peaked on a diet that was slightly carbohydrate-rich and dropped off dramatically on diets with an excess of protein (Lee, Simpson & Raubenheimer 2004). Thus, the main criteria for self-medication are satisfied.

Comparison of overall feeding patterns of virus-challenged and control insects in both experiments suggests that challenged individuals self-medicate on protein, but closer analysis of the feeding dynamics supports a plastic response in which feeding behaviour changes as the viral infection progresses. Among caterpillars that had been given a high (LD_50_) dose of virus, those which survived viral challenge behaved very differently from those that died. The first day post-inoculation was characterized by a sharp increase in P consumption and an elevated P: C ratio in survivors relative to controls and casualties. P and C consumption then declined in survivors over the course of experiment, resulting in a decrease in total food consumption. Note that this dynamic is masked if survivors and casualties are lumped together.

Experiment 2 showed that this change in behaviour was not simply caused by families which naturally choose higher levels of protein being more likely to survive infection. We also tested diet preference *before* infection so that we could be sure that any differences in feeding behaviour were a response to the virus challenge. Prior to inoculation, the digestible component of the diet comprised around two-thirds carbohydrate and one-third protein. In the non-challenged controls, the amount of protein in the diet remained low but gradually increased as pupation approached. In contrast, sublethally infected larvae radically changed their feeding behaviour on a daily basis (Fig. 5), and this is likely to have coincided with temporal changes in the viral infection process (Keddie, Aponte & Volkman 1989; Washburn, Kirkpatrick & Volkman 1996; Cory & Myers 2003). On day 1, there was a dramatic *reduction* in the amount of carbohydrate consumed by the virus-challenged larvae and a decline in the overall feeding rate (Fig. 5b, d). This change in feeding behaviour coincided with the period when virus released from the ingested OBs invades the larval midgut epithelial cells and replicates in their nuclei. Importantly, the amount of protein eaten by inoculated larvae was *maintained* at pre-infection levels, such that the percentage of protein in the diet increased from <40% to *c*. 60% in all of the families we tested. By day 2, carbohydrate intake returned to pre-infection levels in the sublethally infected insects, such that total food consumption increased and the overall P: C ratio declined towards 1: 1. This change in feeding behaviour coincided with a period when many infected midgut cells are likely to have become melanized, encapsulated and/or sloughed into the gut lumen to be replaced by healthy cells, and in some larvae, virus will have migrated into the insect haemocoel to infect haemocytes and other tissues. By day 3, the total food intake of virus-challenged larvae continued to increase, perhaps to offset the reduced food consumption earlier in the infection. Finally, by day 4, the dietary P: C ratio and total food intake of virus-challenged caterpillars became comparable to that of non-infected control larvae, presumably as the infection has been controlled and is no longer imposing a nutritional demand on its host.

Although we detected genetic variation for nutrient consumption, this explained a relatively small amount of the variation in feeding behaviour and was independent of treatment or time post-infection. Rather, diet choice showed a high degree of phenotypic plasticity and different families demonstrated the capacity to respond to infection by self-medicating. Of particular note is that the immediate response following inoculation with a sublethal dose of virus is that the larvae limit their consumption of carbohydrate and food intake overall, but maintain a constant level of protein ingested. This behaviour is consistent with a form of illness-induced anorexia (Kyriazakis, Tolkamp & Hutchings 1998; Adamo, Fidler & Forestell 2007). Specifically, the anorexic response could limit the ingestion of further virus OBs with contaminated food, or it could be a mechanism by the host to reduce calorie intake overall (or carbohydrate intake specifically) without sacrificing protein consumption. Another explanation is that this is the most efficient mechanism by which the host can alter the blend of ingested food to bias it towards proteins; this would be an adaptive response if a protein-rich diet enhances resistance to the virus or limits the virus replication rate.

To explore the impact of macronutrients on possible viral resistance mechanisms, we assayed several aspects of immune function. In both virus-challenged and control larvae, the haemolymph protein pool increased linearly with the amount of protein in the diet. Thus, short-term changes in larval feeding behaviour are reflected in rapid changes in the nutritional composition of their blood (see also Povey *et al*. 2009). The P: C composition of the diet was also reflected in constitutive levels of PO activity, antimicrobial activity and haemocyte density, all three of which increased (linearly or nonlinearly) with increasing protein content of the diet, though unlike the other haemolymph properties, peak antimicrobial activity was not achieved on the most protein-rich diet. This suggests that larvae that switch from a carbohydrate-biased diet onto a diet that is relatively protein-rich will generally have more haemocytes and higher levels of PO with which to melanize and encapsulate virus-infected cells (Washburn, Kirkpatrick & Volkman 1996; Trudeau, Washburn & Volkman 2001), as well as a greater capacity to combat concomitant microbial infections. However, only PO activity and haemocyte density were significantly modulated by viral infection, with virus-challenged larvae having marginally more haemocytes and lower PO activity. Haemocytes are involved in the encapsulation of virus-infected tissues, and so, their greater density in infected larvae may reflect their increased production following infection. The reduction in PO activity in virus-infected larvae is counter-intuitive, but is consistent with previous studies, suggesting phenotypic and genetic trade-offs between immune traits (Cotter, Kruuk & Wilson 2004; Cotter *et al*. 2004; Povey *et al*. 2009; Rao, Ling & Yu 2010). Thus, whilst pre-ingestive behavioural plasticity allows infected individuals to capture the resources required to mount an effective immune response, post-ingestive internal trade-offs may constrain immune expression (Cotter *et al*. 2011). It is also worth noting, however, that other important viral resistance mechanisms have not been quantified in this study, such as the sloughing and replacement of infected midgut epithelial cells, and the resource implications of these processes are not easily quantified.

Finally, this study builds on two previous investigations of the impact of macronutrients on insect resistance to pathogens and the dietary choices insects make when faced with a pathogen challenge (Lee *et al*. 2006; Povey *et al*. 2009). Each study used different host–pathogen combinations, but broadly similar protocols in the same research laboratory, providing the opportunity to explore the generality of their key findings. Lee *et al*. (2006) found that *S. littoralis* larvae challenged with an LD_50_ dose of *S. littoralis* NPV had highest survival on the diet with the highest relative protein content, as also observed here for *S. exempta* and its specific NPV, so demonstrating the importance of protein for resisting baculovirus across different host–virus combinations. Povey *et al*. (2009) conducted a similar experiment using *S. exempta* challenged with the bacterium, *Bacillus subtilis*, suggesting that protein is perhaps ubiquitously important for resisting entomopathogens. This comparison is particularly revealing since the baculovirus infects orally, whereas the bacterium was injected into the haemocoel, suggesting that dietary protein may benefit multiple defence mechanisms in the gut, haemocoel and elsewhere. In diet-choice experiments, *S. littoralis* larvae that were challenged with an LD_30_ dose of baculovirus ate significantly less food post-infection than did the control larvae (Lee *et al*. 2006), so demonstrating a similar anorexic response to that shown by the *S. exempta* larvae receiving an LD_50_ dose of virus in the present study (Experiment 1). Moreover, in both these experiments, larvae that subsequently survived a potentially lethal dose of virus chose a P: C ratio that was significantly more protein-rich than those that succumbed. However, because of the high levels of virus-induced mortality in prior experiments, and the fact that dietary preferences before viral challenge were not quantified, we could not exclude the possibility that these results depended on genetic or other intrinsic differences in dietary preferences of larvae that predisposed them to dying of NPV (Lee *et al*. 2006). Both of these deficiencies were remedied in Experiment 2 of the present study by challenging *S. exempta* larvae with a low dose of virus and by quantifying feeding preferences prior to virus challenge, so that we could monitor shifts in feeding behaviour from pre- to post-infection. These clearly revealed that individuals from different families all switched to a relatively protein-rich diet immediately following infection before returning to a diet that resembled that of control larvae over the following days. It is also worth noting that in none of these experiments did we quantify viral loads in dead or surviving larvae, and so we cannot rule out the possibility that protein-biased diets either alter host tolerance or trigger the virus to switch to a vertically transmitted mode. These possibilities would make interesting avenues for further study.

In conclusion, as predicted, we showed that (i) survival following virus challenge declined as the relative protein content of the diet was reduced; (ii) increasing dietary P: C ratio resulted in higher levels of all immune traits, so providing a potential mechanism for changes in resistance; (iii) when given a choice between complementary diets, virus-challenged insects temporarily increased the relative protein content of their diet, but in insects challenged with a low viral dose, this was achieved by reducing the intake of carbohydrates whilst maintaining protein intake; (iv) infection with a low dose of NPV triggered a short-term anorexic response, so limiting the potential for further exposure to the virus or starving it of key resources. In contrast, we found little evidence for prediction (v), that the degree of plasticity in the ‘self-medication’ response would vary between full-sibling families. Whilst the total amounts of each macronutrient consumed varied between families, the P: C ratio achieved did not, suggesting that this choice is not genetically determined but is a form of phenotypic plasticity common to all genotypes. Our results have clear implications for the foraging behaviour of *S. exempta* larvae in the wild and may help explain the diverse range of graminaceous plant species included in their diet (Yarro 1984; Rose, Dewhurst & Page 2000).

## Data accessibility

Data available from the Dryad Digital Repository: http://dx.doi.org/10.5061/dryad.ps173 (Povey *et al*. 2013).
